# 

**Published:** 1864-12

**Authors:** 


### BOOKS AND PAMPHLETS RECEIVED.

*The Book of Prescriptions, containing* 3000 *prescriptions, collected from the practice of the most eminent Physicians and Surgeons, English, French and American; comprising also a compendious history of the Materia Medica, lists of the doses of all officinal or established preparations, and an Index of Diseases and Remedies. By* Henry Beasley, *author of "The Druggists' Receipt Book" and "The Medical Formulary." Philadelphia:* Lindsay *&* Blakiston, 1865.

*The Functions and Disorders of the Reproductive Organs in Childhood, Youth, Adult Age and Advanced Life, considered in their Physiological, Social and Moral relations. By* William Acton, M. R. C. S., *late Surgeon to the Islington Dispensary, and formerly Externe to the Venereal Hospitals Paris, Fellow of the Royal Med. and Chir. and Statistical Societies, etc., etc. From the last London edition. Philadelphia:* Lindsay & Blakiston, 1865.

*"Spinal Irritation;" or, the Causes of Back-Ache among American Women; from the Transactions of the Medical Society of the State of New York for* 1864. By Charles Fayette Taylor, M. D., *of New York. New York:* Wm. Wood & Co.

